# India Allele Finder: a web-based annotation tool for identifying common alleles in next-generation sequencing data of Indian origin

**DOI:** 10.1186/s13104-017-2556-2

**Published:** 2017-06-27

**Authors:** Jimmy F. Zhang, Francis James, Anju Shukla, Katta M. Girisha, Alex R. Paciorkowski

**Affiliations:** 1 Center for Neurotherapeutics Development, University of Rochester Medical Center, Rochester, NY USA; 2 Rochester Institute of Technology, Rochester, NY USA; 3 Department of Medical Genetics, Kasturba Medical College, Manipal University, Manipal, Karnataka India; 4 Child Neurology, Department of Neurology, University of Rochester Medical Center, 601 Elmwood Avenue, Rochester, NY 14642 USA; 5 Department of Pediatrics, University of Rochester Medical Center, Rochester, NY USA; 6 Departments of Neuroscience and Biomedical Genetics, University of Rochester Medical Center, Rochester, NY USA

**Keywords:** Population genomics, India, Variant annotation, Whole exome sequencing

## Abstract

**Objective:**

We built India Allele Finder, an online searchable database and command line tool, that gives researchers access to variant frequencies of Indian Telugu individuals, using publicly available fastq data from the 1000 Genomes Project. Access to appropriate population-based genomic variant annotation can accelerate the interpretation of genomic sequencing data. In particular, exome analysis of individuals of Indian descent will identify population variants not reflected in European exomes, complicating genomic analysis for such individuals.

**Results:**

India Allele Finder offers improved ease-of-use to investigators seeking to identify and annotate sequencing data from Indian populations. We describe the use of India Allele Finder to identify common population variants in a disease quartet whole exome dataset, reducing the number of candidate single nucleotide variants from 84 to 7. India Allele Finder is freely available to investigators to annotate genomic sequencing data from Indian populations. Use of India Allele Finder allows efficient identification of population variants in genomic sequencing data, and is an example of a population-specific annotation tool that simplifies analysis and encourages international collaboration in genomics research.

## Introduction

Whole exome sequencing (WES) has revolutionized genomic diagnostics and is a key tool in identifying the causal genes underlying rare Mendelian disorders [1–3]. A critical strategy in post-sequencing analysis involves screening a proband’s exome variants against exomes from reference individuals matching the ethnic makeup of the proband. While these data are widely available for individuals from European and African American descent [4, 5], such reference data is less accessible when analyzing exomes from individuals from India. We present India Allele Finder (IAF), an online database table of allele frequencies of individuals from the Indian subcontinent.

The 1000 Genomes web browser (http://www.ncbi.nlm.nih.gov/variation/tools/1000genomes/) effectively presents complete allele frequencies, but rapid queries are more difficult, and annotation of local variant call files (vcfs) is not possible. In contrast, the IAF website and its accompanying command line tool are focused only on the South Indian population, and allow researchers to easily annotate their own exome data sets. Clinicians who want a more ordered method of browsing 1000 Genome data will find the query-based website intuitive to use, while bioinformaticians who work with vcfs will easily adopt the IAF command line tool into their workflow.

## Main text

### Accessing 1000 Genomes data

Fastq data of individuals specific to Indian populations (flagged with “ITU” indicating Indian Telugu ancestry) available via the 1000 Genomes Project [6] were aggregated via ftp from the 1000 Genomes Project, and combined into two fastq files per individual, one per paired end read. We downloaded 100 fastqs out of 118 available ITU individuals from the 1000 Genomes data set. Automated shell scripts facilitated the downloading of fastq files, while an aggregator written in Python concatenated fastqs of the appropriate paired end such that each individual had two fastq files of equal size.

### Data analysis

Fastqs were mapped with the Burrows–Wheeler alignment (BWA) tool 0.7.9a to hg19. The resulting bam files were then analyzed with SAMtools 0.1.19, Picard 1.114, and the Genome Analysis Toolkit (GATK) 3.1.1. Annotation of resulting vcfs was performed with Annovar. A command line Python script, indiaAlleleAnnotator.py, takes as its input a tab delineated vcf and outputs a modified vcf with an additional column representing the allele frequency among the Indian Telugu population.

### Database schema

The vcf generated from the analysis was converted into structured query language (SQL) format, and imported into mysql v.14.14 database as one table. The database is accessed on-line via a Perl Catalyst front-end. The files for this implementation, including the raw SQL file, are available at https://github.com/Paciorkowski-Lab/IndiaAlleleFinder.

**Fig. 1:**
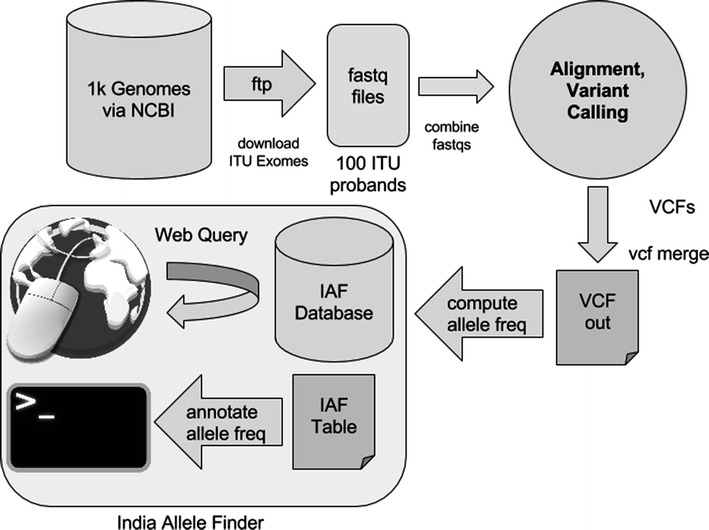
Workflow of analysis of publicly available ITU fastqs from 1000 Genomes used to construct the IAF dataset. Users wishing to annotate exome results with frequency data from IAF may do so using web-based or the command-line interface

IAF allows query of variants through its web-based database, as well as providing a command line tool to annotate exome vcfs. Accepted formats for the web-based query include gene symbol, variant genomic location, or rsID number. The command line annotation tool identifies variants that are present in the IAF data set, and therefore likely to be population variants that may be excluded from further analysis in disease gene identification studies. The IAF workflow is represented in Fig. 1.

## IAF use case study

Subjects MP14-001a1, MP14-001a2, two siblings presenting with achalasia–addisonianism–alacrima syndrome (AAAS), as well as the father and mother, were selected for study. Saliva-derived DNA underwent WES using the Agilent Sure-Select 50 Mb whole exome capture kit, and 100 basepair paired-end reads were generated on an Illumina HiSeq 2500 machine at the University of Rochester Genomics Research Center. Sequence was aligned, analyzed as described previously. De novo, autosomal recessive, and X-linked variants were identified and common variants in the database of single nucleotide polymorphisms (dbSNP) version 137 excluded. We then used IAF to identify and exclude variants found in the 100 Telugu Indian individuals from 1000 Genomes. After filtering by pedigree hypothesis, candidate variants were reduced from 84 to seven when using IAF. We found that MP14-001a1 and MP15-001a2 were homozygous for c.43C>A/p.Q15K variant, a known *AAAS* sequence variation [7]. Their mother and father were both heterozygous for this variant.

The analysis of exome data from populations other than European and African American can be challenging due to difficulty accessing appropriate normal population data sets. This can result in an excess of candidate variants in disease gene identification studies. We have designed IAF to fit into existing workflows.

There are differences between results reported in 1000 Genomes vs IAF. Overall, the IAF data set reports fewer variants, likely due to our use of the newer version GATK v3.1.1 versus v2.4 [8]. Additionally, we sampled from a smaller group of 100 individuals. 1000 Genomes overall collected data from 2535 individuals from 26 different populations for their phase 3 study. As a result, 1000 Genomes aggregated over 5.2 million entries for chromosome 5 alone. Our data set for chromosome 5 contains 8520 entries aggregated from 100 individuals. We anticipate more variants will be represented in IAF as more exomes from the Indian continental population are added.

## Limitations

IAF is a proof of concept implementation of a filtering mechanism based on population-derived variant frequencies. It is a unique tool to further annotate vcfs for the specific purpose of analyzing WES data from individuals of Indian subcontinent descent. We anticipate a proliferation of reference databases for populations that are not of European origin. Additional features are planned for the IAF website, including the ability to input multiple variants, and access a subset of the vcf output corresponding to the genes and/or variants queried. Further exome data sets from individuals of continental Indian ancestry will be added in the future as they become available.

## Abbreviations

AAAS: achalasia–addisonianism–alacrima syndrome
BWA: Burrows–Wheeler alignment tool
dbSNP: database of single nucleotide polymorphisms
GATK: Genome Analysis Toolkit
ITU: Telugu
SQL: structured query language
vcf: variant call file
WES: whole exome sequencing
